# Provost of the University of Pennsylvania

**Published:** 1881-02

**Authors:** 


### EDITORIAL DEPARTMENT.

"If thou hast truth to utter, speak;
And leave the rest to God."

After reposing, for periods more or less brief, the Metric arrangement for prescribing medicines has appeared again in some of the medical periodicals of this country. We had begun to hope it was "tucked away in its little bed" for a quiet nap of indefinite duration, or buried in a grave from which there would be found few, if any, in the United States, willing to undertake its resurrection; but we were doomed to experience disappointment from a quarter we least expected. The usually conservative and appreciative *Record*, has recently come to the front as endorser and advocate of the measure. We have always thought, and tried to practice, on the motto, in medicine as well as in other things, that it was a good plan to let well enough alone; and surely our brother of the *Record*, has not, thus far, brought forward any argument that would cause us to abandon a tried and useful system, for an untried one; or at most, only tried to such an extent as to show its greater complexity, and liability to error, in writing and compounding prescriptions. The universality of its use, is of little weight, when its *greater* liability to error is considered, in the decision of the case; and that it is far more likely to lead to fatal errors and mistakes amongst us, who have been so long accustomed to the old familiar Apothecary's weights and measures, needs no argument to establish, other than a comparison of the expressions and signs used in the two systems. But if a change must be made, let it be for the better, and then invite our confreres beyond the water, to unite with us, in a simple and efficient method, and one that would endure forever, viz: let us adopt a *decimal* system of weights and measures analagous to our unequaled system of dollars and cents?

Has it never occurred to those who seem willing to adopt this metric system for the advantage of our European brethren, for at last that is the *only* real argument, which has been thus far adduced in its favor, that the nations of the old world have pertinaciously adhered to their several systems of coinage and reckoning money, notwithstanding we have been before them, and dealing with them in our *incomparable* decimal system for more than a century? Our mother country with which our interchange of commodities is larger than any other, and we had nearly said, all the other countries on the face of the globe, still reckons her money in pounds, shillings, pence, and farthings, in the face of our *perfect*, and *simple* system of dollars and cents! When a step which so deeply interests the health, lives and comfort of our people is to be taken, let it not be made backward, but forward, and if we must make a change in the doses of medicine, or the names of them, let it not be the metric, but the decimal, system we adopt! Should such a change be thought advisable, the details could be arranged in an hour, and comprehended at a glance, by the merest tyro in medicine. But more hereafter.

PROVOST OF THE UNIVERSITY OF PENNSYLVANIA.—Prof. William Pepper, M. D., has been elected to the honorable and highly responsible position of Provost of this old and distinguished institution. No better man could have been made the chief officer of the University, and from Dr. Pepper's well known intelligence and executive ability, all of us who claim to be sons of the great *Alma Mater*, may confidently look forward to a new era in its already distinguished career. We congratulate the institution, and our worthy confrere, upon the bright future which is now opened up for both of them.

